# Liver and Spleen Stiffness as Non‐Invasive Tools Assessing Liver Congestion in Patients With Budd‐Chiari Syndrome

**DOI:** 10.1111/liv.70870

**Published:** 2026-09-14

**Authors:** Andreea Fodor, Marcos A. Thompson, Oana Nicoara‐Farcau, Ernesto Belmonte, Ángeles Garcia‐Criado, Anna Darnell, Valeria Pérez‐Campuzano, Pamela Vizcarra, Isabel Requejo, Ángeles Falga, Marina Veléz, Irene Fernandez, Adriana Sueiras, Sonia Torres, Jakub Gazda, Anna Baiges, Sarah Shalaby, Asunción Ojeda, Diego San Martin, Bogdan Procopet, Virginia Hernández‐Gea, Fanny Turon, Juan Carlos García‐Pagan

**Affiliations:** ^1^ Barcelona Hepatic Hemodynamic Laboratory, Liver Unit Hospital Clínic, FRCB‐IDIBAPS (Fundació de Recerca Clínic Barcelona‐Institut d'Investigacions Biomèdiques August Pi i Sunyer) Barcelona Spain; ^2^ CIBEREHD (Centro de Investigación Biomédica en Red. Enfermedades Hepáticas y Digestivas). Health Care Provider of the European Reference Network on Rare Liver Disorders (ERN RARE‐Liver). CSUR Centro de Referencia del Sistema Nacional de Salud en Enfermedad Hepática Compleja. Departament de Medicina i Ciències de la Salut Universitat de Barcelona Barcelona Catalonia Spain; ^3^ Gastroenterology Department, 3rd Medical Clinic University of Medicine and Pharmacy ‘Iuliu Hatieganu’ Cluj‐Napoca Romania; ^4^ Radiology Department, CDI, Hospital Clínic de Barcelona Universitat de Barcelona Barcelona Spain; ^5^ 2nd Department of Internal Medicine Pavol Jozef Safarik University and L Pasteur University in Kosice Slovakia

**Keywords:** elastography, hepatic vein thrombosis, portal hypertension, TIPS

## Abstract

**Background and Aims:**

Assessing liver congestion and portal hypertension (PHT) in Budd–Chiari syndrome (BCS) remains challenging. We evaluated liver and spleen stiffness measurements (LSM, SSM) obtained by transient elastography as non‐invasive tools for identifying clinically meaningful liver congestion in BCS.

**Methods:**

This retrospective study included consecutive patients with BCS followed at our unit. At each visit, patients were classified as having a controlled or uncontrolled clinical status based on clinical, biochemical, imaging and/or hemodynamic data, by absence or presence of clinically significant PHT. LSM and SSM were independently obtained at all visits and diagnostic performance for classifying clinical status was assessed using receiver operating characteristic (ROC) analysis.

**Results:**

Sixty‐six patients were included with a median follow‐up of 65.5 months (range:2–182): 34 had persistently controlled status, 11 persistently uncontrolled, 14 initially uncontrolled improved to sustained controlled status and 7 showed fluctuating status. Two‐hundred eighty‐nine LSM were obtained during periods of controlled clinical status (median:15.1 kPa, IQR:10.9–19.9) and 203 during uncontrolled status (median: 41.1 kPa, IQR: 34.8–64.5). LSM discriminated controlled from uncontrolled status (AUC 0.985). LSM cut‐off of 25 kPa yielded 97.5% sensitivity, 92.5% specificity, 97.6% negative predictive value and 90% positive predictive value. SSM showed good discrimination (AUC 0.854), with values ≤ 30 kPa ruling out and > 40 kPa ruling in uncontrolled status, but accuracy was inferior to LSM. LSM values rapidly decreased following liver congestion resolution after interventional procedures.

**Conclusions:**

In BCS, LSM shows excellent concordance with clinical status and periodic assessment may facilitate clinical management. SSM provides complementary information but does not outperform LSM.

## Key Points

- Longitudinal liver stiffness measurements closely reflected changes in clinical status in patients with Budd–Chiari syndrome.
- A liver stiffness threshold of 25 kPa distinguished controlled from uncontrolled status with 97.5% sensitivity, 92.5% specificity and 97.6% negative predictive value.
- Serial liver stiffness measurement may offer a simple, non‐invasive adjunct for monitoring patients with Budd–Chiari syndrome over time.
- Spleen stiffness provided complementary information but showed lower diagnostic performance and was influenced by myeloproliferative neoplasms and portal venous axis thrombosis.

## Lay Summary

Monitoring disease severity in Budd–Chiari syndrome remains difficult. We found that liver stiffness closely mirrored patients' clinical status and decreased after successful treatment relieving liver congestion. Repeated liver stiffness measurements may provide a simple, non‐invasive way to follow patients over time.

**Abbreviations**
ACO: anticoagulation
BCS: Budd–Chiari Syndrome
CSPH: clinically significant portal hypertension
IR: interventional radiology
IVC: inferior vena cava
LSM: liver stiffness measurement
LT: liver transplant
MPN: myeloproliferative neoplasm
PHT: portal hypertension
PPG: portal‐cava pressure gradient
PVT: portal vein thrombosis
SSM: spleen stiffness measurement
TE: transient elastography
TIPS: transjugular intrahepatic portosystemic shunt

## 1 Introduction

Budd–Chiari Syndrome (BCS) is a rare disease caused by the obstruction of hepatic venous outflow that can occur from small hepatic venules to the inferior vena cava (IVC) leading to liver congestion and ischemic hepatic injury [1]. Liver congestion is the main factor leading to the development of portal hypertension (PHT) and, as in other liver diseases, the development of PHT may result in severe, potentially life‐threatening complications.

Current management of BCS follows a stepwise approach, progressively escalating the invasiveness of treatment (anticoagulation to transjugular intrahepatic portosystemic shunt—TIPS and ultimately liver transplantation—LT) according to the evolution of the patients [1]. However, this approach remains difficult to standardize and largely relies on individualized, case‐by‐case discussion because, although different specific scores have been developed to classify the severity of BCS, none of them is accurate enough to guide treatment and reliably predict the outcome [2, 3].

In patients with cirrhosis, liver stiffness measurement (LSM) has been extensively validated as a non‐invasive tool for staging liver fibrosis and assessing the severity of PHT [4, 5]. In patients with BCS and PHT‐related complications, LSM has been proposed as a tool to stratify disease severity and assess the efficacy of interventional treatments [6, 7, 8, 9]. Indeed, an increase in LSM of 44.7% or ≥ 8 kPa during follow‐up has been suggested as a non‐invasive marker of recurrent liver congestion, potentially indicating TIPS dysfunction [10]. However, these cut‐off values have not been validated so far and the role of LSM in the longitudinal monitoring of BCS remains to be established.

Spleen stiffness measurement (SSM) has been shown to improve the non‐invasive diagnosis of clinically significant portal hypertension (CSPH) in patients with advanced chronic liver disease [11, 12, 13], as well as in patients with vascular disorders of the liver such as in porto‐sinusoidal vascular disorder [14].

This study aimed to evaluate the value of LSM and SSM as non‐invasive markers of liver congestion, as a surrogate of portal hypertension, in patients with BCS and to assess their potential utility for monitoring response to treatments.

## 2 Methods

### 2.1 Study Design and Patient Inclusion

We conducted a retrospective, single‐centre, observational study including all consecutive patients with BCS who were either under active follow‐up or newly diagnosed between 2019—when LSM was incorporated into routine clinical evaluation and follow‐up visits of the patients with vascular liver diseases at our centre—and March 2023. At our centre, LSMs are performed by trained nurses in a dedicated hospital area and recorded using a standardized reporting form. Patients also underwent a comprehensive clinical evaluation by hepatologists from the team, laboratory testing and an abdominal ultrasound: (a) routinely every 6 months; or (b) earlier, when a scheduled medical visit was advanced due to clinical deterioration, including worsening general condition, abdominal distension, lower limb oedema or decompensation of PHT in the form of ascites, variceal bleeding or hepatic encephalopathy. In patients newly diagnosed with BCS, LSM was performed at increased frequency according to clinical evolution. Until March 2022, LSM was the only elastography technique performed. From that date onward, SSM was incorporated into the clinical workflow and has since then been performed simultaneously with LSM by the same trained nurses.

### 2.2 Liver and Spleen Stiffness Measurements

LSMs were obtained by transient elastography (TE) using a Fibroscan 502 (prior to 2022) or a Fibroscan Expert (from 2022 onward), both by Echosens, Paris, France. SSMs were obtained using the dedicated spleen probe of the Fibroscan Expert. Studies were performed according to the manufacturer's instructions and guidelines by trained nurses with over 5 years of experience, that had manufacturer's certification for performing LSM and SSM studies. A LSM value was considered valid if the success rate of 10 measurements was > 60% and the IQR/Median was < 30%. In patients with ascites in whom LSM/SSM measurements were initially unsuccessful, the assessments were repeated after a large‐volume paracentesis.

### 2.3 Definitions and Patient Group Classification

The clinical records of all patients were retrospectively reviewed by two independent investigators (either AF and VPC or MT and OF) to classify the clinical status of each patient at every study visit as either controlled or uncontrolled. In the absence of validated definitions of clinical status in BCS, patients were categorized into two study‐specific states based on objective manifestations of clinically relevant hepatic congestion. Importantly, classification was performed independently of LSM and SSM values, thereby avoiding incorporation bias.

A patient was considered to have controlled BCS when, under the current therapeutic strategy, the patient remained free of clinically significant liver‐related complications and showed no evidence of disease progression or treatment failure requiring escalation to the next step of the therapeutic algorithm. Specifically, controlled disease was defined by the absence of ascites, variceal bleeding or other clinically significant complications of portal hypertension; stable or improving liver function (including bilirubin, INR, albumin and MELD/Clichy scores); preserved renal function; no radiological evidence of worsening hepatic venous outflow obstruction; and no need for TIPS placement, additional endovascular intervention or liver transplantation at the time of assessment.

Patients were considered free of ascites only in the absence of ascites on abdominal ultrasound and in the absence of ongoing diuretic therapy prescribed for ascites. In patients who had undergone TIPS placement, classification as controlled additionally required the absence of hemodynamic or ultrasound evidence of TIPS dysfunction (see below).

Conversely, patients were classified as having uncontrolled BCS if one or more criteria for controlled disease were not fulfilled. In addition, it is our clinical practice to perform an upper gastrointestinal endoscopy in patients for whom there was clinical uncertainty during follow‐up regarding the potential need for further therapeutic intervention. Patients found to have high‐risk varices were retrospectively classified as having uncontrolled disease.

Discrepancies between reviewers regarding clinical status classification were resolved through joint re‐evaluation and discussion with JCGP and FT, after which a final consensus was reached.

For the purpose of treatment‐based evaluation, patients were also categorized into two groups according to the therapeutic strategy in place at each time point: the anticoagulation group (ACO group) and the interventional radiology group (IR group). The IR group included patients undergoing suprahepatic vein angioplasty, suprahepatic vein stent placement, TIPS insertion or a combination of these procedures.

Both clinical status and treatment group were therefore considered dynamic variables, with the possibility of changing at successive evaluations.

Because, in our experience, successful IR procedures usually produce rapid changes in LSM that tend to stabilize after 3 months, the LSM/SSM values obtained during this period were not assigned to either controlled or uncontrolled clinical status but were analysed separately to assess post‐treatment changes.

#### 2.3.1 TIPS Dysfunction Definitions

Hemodynamic TIPS dysfunction: direct measurement of the porto‐cava pressure gradient (PCG) showing ≥ 12 mmHg.

Ultrasound TIPS dysfunction: one of the following: a mean of the maximum portal blood flow velocity of less than 28 cm/s together with a hepatofugal flow direction in the intrahepatic portal vein branches or a mean of max portal blood flow velocity of less than 39 cm/s when flow in the intrahepatic portal vein branches is hepatopetal or the presence or persistence of ascites [15].

### 2.4 Statistical Analysis

Continuous variables are presented as median (interquartile range [IQR]) and categorical variables as number (percentage). Data distribution was assessed using the Shapiro–Wilk test. Comparisons between two groups were performed using the independent‐samples Student's *t*‐test or the Mann–Whitney U test, as appropriate according to data distribution.

Receiver operating characteristic (ROC) curves were generated to differentiate between clinical controlled and uncontrolled status and the area under the ROC curve (AUROC) was calculated. An appropriate cut‐off was obtained from the curve based on the sensitivity and specificity using the Youden index. A 2‐tailed *p* value < 0.05 was considered statistically significant. Statistical analysis was performed using SPSS 23.0 (SPSS inc., Chicago, IL, USA).

In addition, we checked in our cohort the potential value identifying liver congestion of a recently described cut‐offs of absolute and percentual variation of LSM. The percentage variation of LSM was calculated as previously described [13] (Current LSM‐Previous LSM/previous LSM) × 100%.

The study was performed in accordance with the ethical guidelines of the Declaration of Helsinki and was approved by the Ethics Committee of Hospital Clinic de Barcelona (HCB/2021/0239). All study participants provided written informed consent.

## 3 Results

### 3.1 Study Population

Sixty‐six patients with BCS were included in the study. Baseline characteristics at inclusion are summarized in Table 1. Median age at inclusion was 40 (IQR 35–52) years. Thirty‐four patients (51.5%) had PHT‐related complications, either prior or at the time of inclusion. The most frequent complication was ascites (either mild or requiring paracentesis). High‐risk oesophageal varices were described in 5 patients, while one presented with gastric varices. Thrombosis of the portal venous system was observed in 17 patients (25.7%). Myeloproliferative neoplasms were the most frequent underlying prothrombotic condition (*n* = 30).

**TABLE 1:** Baseline characteristics and associated disorders. Values are expressed as number (percentage %) or median (IQR 25–75).

Characteristic	*n* (%)/median (IQR)
Female gender	46 (69.7%)
Age at inclusion	40 (35–52)
Underlying conditions	
Myeloproliferative neoplasm	30 (45.4%)
Antiphospholipid syndrome	7 (10.6%)
Factor V Leiden mutation	4 (6.0%)
Paroxysmal hemoglobinuria	1 (1.5%)
Protein S deficiency	1 (1.5%)
Prothrombin deficiency	1 (1.5%)
Systemic diseases	3 (4.5%)
Laboratory data at inclusion	
ASAT (U/L)	34 (29–73)
ALAT (U/L)	32 (21–39)
Total bilirubin (mg/dL)	1.3 (0.7–2.2)
ALP (U/L) (normal range < 116 U/L)	183 (143–329)
GGT (U/L) (normal range < 40 U/L)	95 (46–159)
Creatinine (mg/dL)	0.74 (0.63–0.89)
INR	1.34 (1.18–1.62)
Albumin (mg/dL)	41 (36–44)
Ascites	25 (37.8%)
High‐risk oesophageal varices	5 (7.6%)
Associated splenoportal axis thrombosis	17 (25.7%)
IR group at baseline	28 (42.4%)
TIPS	25
Angioplasty only	2
Angioplasty plus stent	1

Abbreviations: IR, interventional radiology; TIPS, transjugular intrahepatic portosystemic shunt.

At the time of inclusion, 28 patients with BCS (42.4%) had already received IR treatment (25 TIPS insertions, 2 angioplasties of short hepatic stenosis and 1 angioplasty with stent placement), while the remaining 38 patients with BCS did not.

The median duration of follow‐up was 65.5 (range 2–182) months and the median time eluded from the diagnosis of BCS to the inclusion of the study (first LSM) was 20 (range 0–297) months.

As shown in Figure S1, among the 66 patients included, 34 were classified within a controlled clinical status at inclusion that remained throughout all study visits (21 treated with ACO and 13 with IR) while the remaining 32 patients were at an uncontrolled status at inclusion. Among these patients, 14 improved and achieved a sustained controlled status during follow‐up (3 on ACO and 11 after IR), 11 remained persistently uncontrolled during the study period (5 treated with ACO and 6 with IR) for different reasons (Table S1), while the remaining 7 patients (3 treated with ACO and 4 with IR) exhibited alternating changes in clinical status (due to rethrombosis and/or TIPS dysfunction). Thus, at least one controlled clinical status was observed in 55 patients and at least one uncontrolled clinical status was observed in 32 patients (Figure S1).

### 3.2 Liver Stiffness Measurement as a Tool for Assessing Liver Congestion in BCS

**FIGURE 1:**
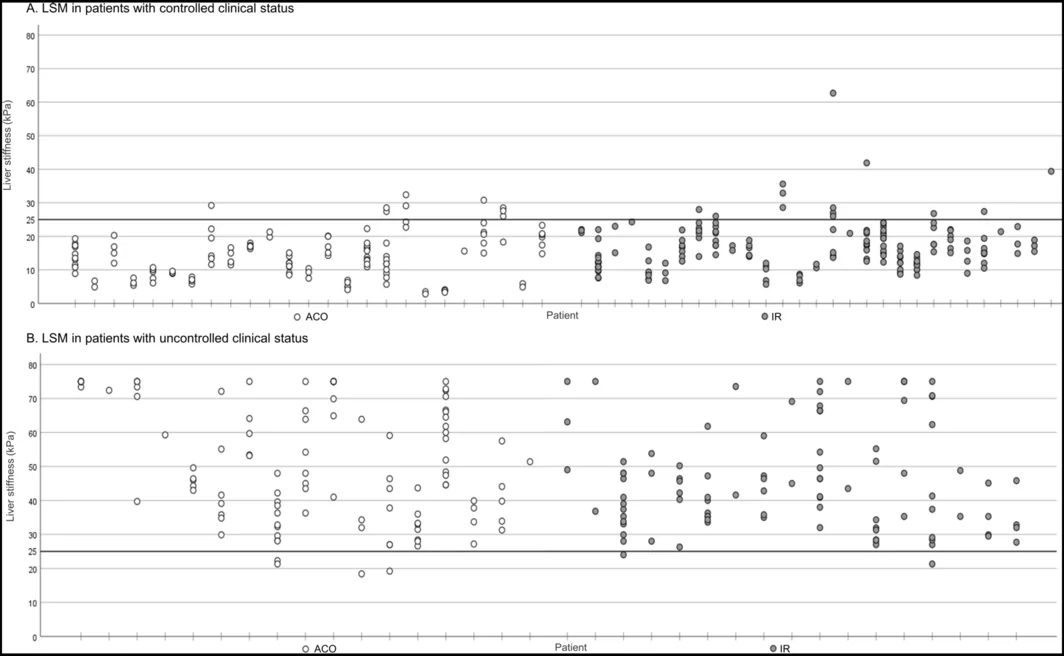
Distribution of LSM values in patients with controlled (A) and uncontrolled (B) clinical status, stratified by treatment (ACO, IR). Horizontal line at 25 kPa indicates the proposed cut‐off for LSM; values > 25 kPa rule in liver congestion.

Among 496 attempted examinations, LSM could not be obtained in four cases (0.8%) due to persistent ascites. Consequently, 492 valid LSM measurements were available and included in the analysis. Overall, 289 LSM were obtained at time points when patients were classified as having a controlled clinical status (Figure 1A), whereas 203 LSM were obtained during periods of uncontrolled clinical status (Figure 1B).

Patients remained in a controlled clinical status for a median of 79 months (IQR 35–117; range 7–149). During this period, a median of 5 LSM per patient were recorded (range 1–15), with a median LSM value of 15.1 kPa (IQR 10.9–19.9). LSM values did not differ significantly according to the presence of an underlying myeloproliferative neoplasm (MPN) (15.1 kPa [IQR 10.9–18.8] vs. 15.3 kPa [IQR 10.3–21.0] in patients without MPN; *p* = 0.5), nor according to the presence of portal or splenic vein thrombosis (16.9 kPa [IQR 13.1–20.9] vs. 14.3 kPa [IQR 10.2–19.5] in those without thrombosis; *p* = 0.5).

Similarly, the median duration of uncontrolled clinical status during which LSM were obtained was 52 months (IQR 29–97). During this period, a median of 6 LSM measurements per patient were available (range 1–8), with a median LSM value of 41.1 kPa (IQR 34.8–64.5). Again, no significant differences in LSM values were observed according to the presence of MPN (45.0 kPa [IQR 35.5–61.9] vs. 45.0 kPa [IQR 33.7–75.0] in patients without MPN; *p* = 0.9) or portal or splenic vein thrombosis (47.4 kPa [IQR 35.8–55.1] vs. 50.2 kPa [IQR 35.0–69.1] in those without thrombosis; *p* = 0.7).

#### 3.2.1 ROC Curve Analysis of LSM for Clinical Status Classification

ROC curve analysis demonstrated excellent discrimination between LSM and clinical status classification (AUROC = 0.985). The Youden index identified an optimal cut‐off of 26.3 kPa (sensitivity 97%, specificity 94%). For clinical use, a 25 kPa threshold was selected (Figure 1A,B), showing comparable performance (sensitivity 97.5%, specificity 92.5%, NPV 97.6%, PPV 90%).

Among patients with a controlled clinical status, 13 individuals contributed 22 LSM measurements above the 25 kPa threshold. In eight patients, this consisted of a single isolated measurement, with all subsequent LSM values below 25 kPa. Of the remaining five patients, three treated with anticoagulation alone showed transiently elevated LSM values that later normalized, whereas two patients treated with IR procedures exhibited persistently elevated LSM values for more than 3 months after TIPS placement despite controlled clinical status (PPG 10 and 4 mmHg, respectively).

Similarly, five patients with uncontrolled clinical status showed LSM values below 25 kPa. In four of them, repeated measurements later exceeded the threshold. The remaining patient had a single LSM value below 25 kPa despite angiographically confirmed TIPS thrombosis.

To mitigate the impact of multiple measurements per patient, a patient‐level analysis including only the first LSM per clinical status was performed, yielding an AUC of 0.983. Using the 25 kPa cut‐off, sensitivity was 100%, specificity 88.2%, NPV 100% and PPV 85%.

We next evaluated the ability of the previously proposed threshold (defined as an increase in two consecutive LSM by more of 44% or ≥ 8 kPa) to identify IR failure. Among 30 patients treated with IR, 191 paired measurements were available. Although the proposed threshold demonstrated high sensitivity (81.8%), specificity (92.1%) and an excellent negative predictive value (98.3%) for ruling out IR dysfunction, its positive predictive value was limited (47.4%). These findings indicate that, while the threshold performs well for excluding IR dysfunction, it is suboptimal for predicting its development.

### 3.3 Spleen Stiffness Measurement as a Tool for Assessing Liver Congestion in BCS

A subgroup of 49 patients (74.2%) underwent SSM concomitantly with LSM. A total of 78 SSM were available from 39 patients who had at least one evaluation classified as having a controlled clinical status. The median number of SSM per patient was 1 (range 1–6) and the median SSM value was 26.9 kPa (IQR: 21.9–36.6 kPa). In contrast, 50 SSM were available from 19 patients with at least one evaluation classified as having an uncontrolled clinical status. In this group, the median number of SSM per patient was 2 (range 1–7) and the median SSM value was 49.3 kPa (IQR: 41.7–64.0 kPa).

**FIGURE 2:**
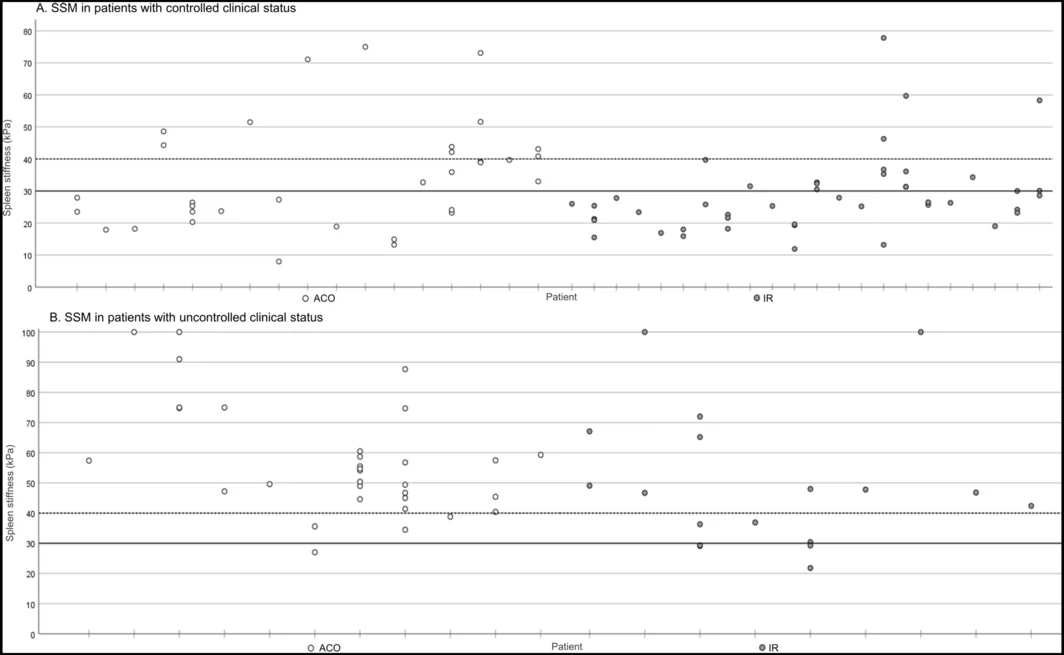
Distribution of SSM values in patients with controlled (A) and uncontrolled (B) clinical status, stratified by treatment (ACO, IR). Horizontal line at 30 kPa cut‐off value to rule out liver congestion. Dotted horizontal line at 40 kPa to rule in liver congestion.

ROC curve analysis demonstrated good discrimination of SSM for clinical status classification (AUC of 0.854). The Youden index identified an optimal cut‐off of 44.6 kPa, yielding a sensitivity of 72% and a specificity of 87%. As illustrated in Figure 2A, SSM ≥ 40 kPa is infrequent in patients with controlled clinical status. Conversely, Figure 2B shows that SSM values ≤ 30 kPa provide a high sensitivity (90%) for ruling out an uncontrolled clinical status. Because SSM values may be influenced by the presence of an underlying MPN or by concomitant portal venous axis thrombosis, potentially leading to portal hypertension despite complete hepatic decongestion, these factors were considered in further analysis. Among patients with controlled clinical status, median SSM values were significantly higher in those with underlying MPN (*n* = 17 patients and 30 SSM) compared with those without MPN (*n* = 22 patients and 48 SSMs): 35.8 kPa (IQR 25.6–45.3) versus 25.2 kPa (IQR 19.6–32.9), *p* = 0.002. Similarly, patients with portal or splenic vein thrombosis (*n* = 11 patients and 21 SSMs) had significantly higher SSM values than those without thrombosis (28 patients and 57 SSMs): 38.9 kPa (IQR 25.3–51.5) versus 26.0 kPa (IQR 20.9–31.5); *p* = 0.008.

Among patients with an uncontrolled clinical status, no significant differences in median SSM values were observed between those with (5 patients, 12 SSM assessments) and without MPN (14 patients, 38 SSM assessments): 52.7 kPa (IQR, 42.7–75.0) versus 49.3 kPa (IQR, 41.7–59.2), *p* = 0.56. Regarding portal vein thrombosis (PVT), only one patient with uncontrolled status had available data (SSM 42.4 kPa), precluding comparison with patients without PVT (18 patients, 49 SSM assessments; median 49.4 kPa, IQR 41.4–65.2). A revised ROC curve analysis evaluating the ability of SSM to discriminate between controlled or uncontrolled clinical status after exclusion of confounding factors (MPN and PVT) yielded an AUROC of 0.91, indicating a modest improvement compared with the initial analysis (AUROC = 0.854). The optimal SSM cut‐off identified by the Youden index was 34.5 kPa, with a sensitivity of 91.9% and a specificity of 80.5%. Despite this improvement, the discriminatory performance of SSM remained lower than that observed for LSM. We next assessed whether concomitant SSM could allow reclassification in patients whose clinical status was misclassified by LSM. However, only one patient with a false‐positive LSM result and without underlying MPN or porto‐splenic thrombosis had concomitant SSM data available. This patient had three LSM > 25 kPa, with corresponding SSM values of 30, 32 and 32 kPa.

### 3.4 LSM as a Tool to Evaluate Treatment Response

**FIGURE 3:**
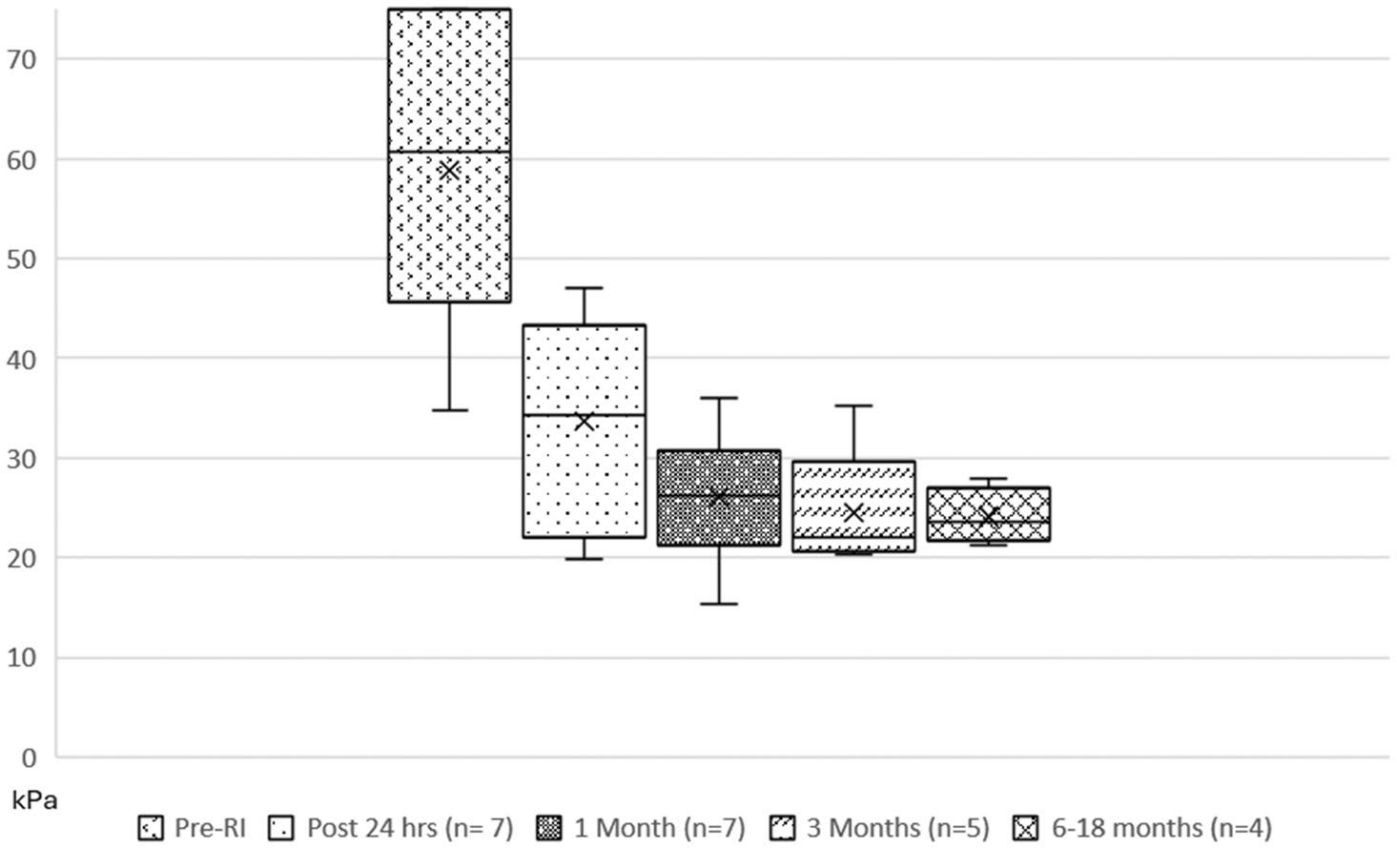
Dynamics of LSM at baseline, 24 h and 1, 3, 6–18 months following IR treatment. The number of patients with LSM performed at each time is clarified between brackets. Box plots represent interquartile range, with the median value represented by the horizontal line.

In 13 patients, LSM was available both before and after achieving a clinically controlled status (three treated with anticoagulation and 10 with IR). Among the three patients who achieved a controlled clinical status with ACO, median LSM decreased from 63.9 kPa (45.4–69.4) before ACO initiation to 24.3 kPa (18.3–26.7) at the time controlled status was first attained, after a median treatment duration of 41 months (range 15–52). All three patients remained in a controlled clinical status throughout the subsequent 2‐year follow‐up. In the 10 patients treated with IR, median pre‐IR LSM was 59.3 kPa (IQR 45.1–75 kPa), which significantly decreased to 33.6 kPa 24 h after the procedure (IQR 26.7–41.5 kPa; *p* = 0.006), corresponding to a median absolute reduction of 23.4 kPa (IQR 13.8–28.2 kPa) or 40% (IQR 30.5%–42.8%). At 1 month, median LSM further decreased to 25 kPa (IQR 21.8–29.5 kPa; *p* = 0.014), representing a median absolute reduction of 38.6 kPa (IQR 15.8–45.3) or by 59% (IQR 45.4%–65.3%). Reduction in LSM was sustained for as long as patients remained in clinically controlled status (Figure 3).

### 3.5 SSM as a Tool to Evaluate Treatment Response

Two patients without MPN or portal/splenic vein thrombosis had SSM before and after achieving a controlled status with IR. Patients have been followed for 3 and 12 months, respectively. Values of pre‐IR SSM were 59.7 and 100 kPa, respectively. In the patient with SSM of 100 kPa, it decreased to 37.3 kPa 24 h after IR, remained stable with 38.9 at 1 month and decreased to 23.4 kPa at 3 months. In the other patient, SSM decreased to 36.1 kPa at 1 month, to 22.1 at 3 and it was 31.3 kPa at 12 months.

## 4 Discussion

LSM by transient elastography has been extensively studied as a surrogate marker for hepatic fibrosis and as a prognostic tool in patients with chronic liver diseases, in some cases replacing invasive methods of risk assessment like the measurement of the hepatic venous pressure gradient. Different publications have shown that an increase in LSM is also observed when liver congestion is present, including in patients with BCS, irrespective of the grade of fibrosis [11, 12, 13].

Furthermore, two recent studies have demonstrated that the relief of liver congestion following a successful radiological procedure (such as TIPS or hepatic vein stent placement or angioplasty) results in a reduction of LSM [11, 12, 13]. Nevertheless, these studies were limited by small sample sizes and relatively short follow‐up periods, leaving uncertainty regarding the clinical utility of LSM for longitudinal assessment of disease status in BCS.

More recently, SSM has shown an important role in predicting portal hypertension‐related events in patients with chronic advanced liver diseases^14‐167^. A small study also suggested a possible role of SSM in the evaluation of portal hypertension in patients with BCS [9]. Therefore, we conducted a retrospective single‐centre study to evaluate the potential role of LSM and SSM as a non‐invasive tool for assessing clinical status in patients with BCS and to explore their performance in relation to treatment‐induced changes and relevant confounding factors.

Our study confirms that LSM could be used as a non‐invasive tool to assess the severity of liver congestion and portal hypertension in patients with BCS [8, 9, 11, 12, 13]. Changes in LSM appear to sensitively reflect variations in the degree of liver congestion. Indeed, following successful hepatic decompression through IR, an immediate reduction in LSM of up to 40% was observed. This early decrease was followed by a further significant reduction within the first 1–3 months and subsequent stabilization over time in patients who achieved and maintained a controlled clinical status. These findings are consistent with recent studies showing stabilization of LSM values in patients who remained with adequate liver decompression within 3–6 months after the intervention [1, 13]. Although only three patients in our cohort had LSM available both before and after achieving clinical stabilization under anticoagulation therapy alone, our observations suggest that liver decongestion in this context occurs more gradually. Consequently, the absence of rapid LSM reduction in LSM following anticoagulation initiation should not be interpreted as evidence against the possibility of a subsequent controlled clinical course. Nevertheless, these findings require confirmation in larger groups of patients treated exclusively with anticoagulation.

Our study provides a unique opportunity to evaluate longitudinal changes in LSM across different clinical scenarios. We observed that most patients who achieved or maintained a controlled clinical status after treatment (anticoagulation or IR) had LSM values ≤ 25 kPa. This threshold showed a strong ability to rule in or rule out persistent or recurrent liver congestion.

In this study, LSM demonstrated excellent concordance with independently assessed clinical classification. Our data suggest that serial LSM may complement clinical assessment during follow‐up. In patients with TIPS, LSM values exceeding 25 kPa during follow‐up may raise suspicion of TIPS dysfunction or recurrent thrombosis and could prompt more invasive hemodynamic assessment. Conversely, persistently low LSM below 25 kPa was rarely associated with clinically significant congestion, suggesting that in this setting, unnecessary invasive investigations could be avoided. Although our study was not designed to assess prognostic outcomes, these findings suggest that serial LSM measurement may assist in longitudinal monitoring by identifying patients in whom persistent liver congestion should be suspected, even in the absence of clinical signs of worsening portal hypertension and may warrant further evaluation.

Similar results were observed in an analysis restricted to the first LSM recorded per patient within each clinical status, suggesting that the findings were not driven by repeated measurements from the same individuals. Whether incorporation of serial LSM into routine follow‐up improves clinical decision‐making should be established in prospective studies.

A recent prospective study assessed the ability of LSM to predict the recurrence of liver congestion, specifically related to TIPS dysfunction. The study found that an increase in LSM of 8 kPa or more, corresponding to 44.7% above the previous value, had a sensitivity of 80% [13]. However, when applied to our cohort, these relative cut‐off values showed lower diagnostic accuracy than absolute LSM thresholds, which appear to be more precise and easier to implement in routine clinical practice (not need to know or have previous LSM values).

Our study also shows the potential utility of SSM in evaluating the clinical status of patients with BCS. However, its agreement with the classification on controlled or uncontrolled status was lower than that of LSM. As demonstrated by our data, there are several confounding circumstances, such as the underlying presence of a MPN or an associated thrombosis of the portal venous axis that could influence SSM even in the absence of liver congestion. This effect was especially evident when no liver congestion was observed and low SSM values would otherwise be expected. However, even after excluding these patients, the diagnostic capacity of SSM remained lower than that of LSM.

This study has several limitations. In the absence of a standardized or widely accepted longitudinal classification of disease status in BCS, patients were classified using study‐specific categories reflecting the presence or absence of clinically meaningful liver congestion. To minimize the risk of misclassification, we implemented several measures. Clinical status was independently assessed by two investigators, with any disagreements resolved by consensus with a third senior investigator. Furthermore, investigators were blinded to the corresponding LSM and SSM results during clinical status assessment. Taken together, we consider the risk of misclassification to be low. In addition, due to the retrospective nature of the study, we could not analyse the variability of LSM at standardized time periods during follow‐up. Although only examinations fulfilling predefined quality criteria were included and LSM/SSM were performed after large‐volume paracentesis when required, measurement reliability may still have been affected in some patients with ascites. The overall number of patients was also limited. However, BCS is a rare disease with low incidence. Our cohort includes a substantial number of longitudinal LSM determinations, with a very high degree of concordance with independently assessed controlled or uncontrolled clinical status, supporting the robustness of our findings. We did not have SSM concomitantly with all the LSM values because we incorporated the spleen dedicated probe in 2022 and it is possible that the results of SSM might improve with a larger sample size that would allow us to correct for the observed confounding factors (underlying MPN and associated portal/splenic vein thrombosis). In addition, the low number of misclassified patients, having both determinations and that do not have confounding factors for SSM does not allow us to assess the potential utility of the combination of LSM and SSM. Therefore, additional data on the potential role of SSM, both alone and in combination with LSM, may be needed. However, given the high accuracy of LSM, the additional value provided by SSM is likely to be limited.

In conclusion, LSM demonstrates an important role as a non‐invasive tool for assessing the severity of portal hypertension and liver congestion in patients with BCS. LSM values below 25 kPa are suggestive of a controlled clinical status and adequate hepatic decompression, whereas values above the 25 kPa threshold are highly suggestive of the presence or recurrence of significant liver congestion. SSM, although it may help assess liver decongestion, exhibits lower diagnostic performance than LSM and is influenced by other conditions, such as the presence of MPN or portal venous axis thrombosis.

## Author Contributions

Data collection and procedures: A.F., M.A.T., O.N.‐F., E.B., Á.G.‐C., A.D., V.P.‐C., P.V., Á.F., S.T., J.G., B.P., J.C.G.‐P., F.T., A.B., I.R., S.S., A.O., D.S.M., V.H.‐G.; concept and design: A.F., M.A.T., O.N.‐F., F.T., J.C.G.‐P.; supervision and final draft: F.T. and J.C.G.‐P.; drafting the article: A.F., M.A.T., O.N.‐F.

## Funding

This work was supported by: FIS PI23/01102 and FIS 23/00997 funded by ‘Instituto de Salud Carlos III’ and co‐funded by the European Union. From ‘CIBEREHD CB06 04 0026’ funded by ‘Instituto de Salud Carlos III’. From the ‘Commissioner for Universities and Research from the Department of Economy and Knowledge’ of the ‘Generalitat de Catalunya’ (AGAUR SGR2021 01115). A.F. is a recipient of the PNRR/2022/C9/MCID/I8‐CF36 grant funded by the Romanian Ministry of Research, Innovation and Digitalization, titled ‘Translational research targeting fibrosis regression, endothelial dysfunction reversal and hepatocyte regeneration in advanced liver diseases – 3RE‐VALID’, under Funding Contract No. 760064/23.05.2023. A.B. is the recipient of a Juan Rodés JR20/0024 grant. P.O. has a Rio Hortega grant; current contract is funded by ‘Instituto de Salud Carlos III’ with charges to the European funds of the Recovery, Transformation and Resilience Plan (Plan de Recuperación, Transformación y Resiliencia), with file code CM22/00058, pursuant to the Resolution of the Instituto de Salud Carlos III, O.A., M.P. of December 14, 2022, granting the Rio Hortega Contracts and ‘Financed by the European Union – NextGeneration EU. S.S. has a Rio Hortega Grant. Current contract is funded by “Instituto de Salud Carlos III”’ with charges to the European funds of the Recovery, Transformation and Resilience Plan (Plan de Recuperación, Transformación y Resiliencia), with file code CM23/00068, pursuant to the Resolution of the Instituto de Salud Carlos III, O.A., M.P. of December, 2023, granting the Rio Hortega Contracts and ‘Co‐Financed by the European Union’.

## Conflicts of Interest

J.C.G.‐P. advisory for GORE, Cook and grant from Mallinckrodt and Astra‐Zeneca. Speaker fees from GORE and Cook. The remaining authors declare no conflicts of interest that pertain to this work.

## Supporting information


**Figure S1:** Distribution and dynamics of clinical status during follow‐up, accounting for LSM and treatment (ACO vs. IR).

**Table S1:** Outcomes of patients with persistently unfavourable clinical status (*n* = 11).

## Data Availability

The data that support the findings of this study are available from the corresponding author upon reasonable request.
