# Supplementary material for: Liver and Spleen Stiffness as Non‐Invasive Tools Assessing Liver Congestion in Patients With Budd‐Chiari Syndrome
Source: Liver Int. 2026 Sep 14;46(10):e70870. doi: 10.1111/liv.70870 (PMC13575439; doi:10.1111/liv.70870)
Supplement: Supplementary file 1 [file LIV-46-0-s002.docx]

Supplementary Table 1: Outcomes of patients with persistently unfavorable clinical status (n=11).

| Treatment strategy | Number of patients | Clinical course and outcomes |
| --- | --- | --- |
| Anticoagulation | 5 | - Lost of follow-up (n=3); - Cerebral hemorrhage with severe neurological sequelae (n=1); - Scheduled for TIPS at the end of study follow-up (n=1). |
| Interventional radiology | 6 | - Lost of follow-up (n=2); - Persistent PPG > 16 mmHg without further decompensation and received beta-blockers (n=1); - Liver transplantation (n=1) - Death (n=2: cerebral hemorrhage, drug overdose) |
